# Establishing and characterizing human stem cells from the apical papilla immortalized by hTERT gene transfer

**DOI:** 10.3389/fcell.2023.1158936

**Published:** 2023-05-22

**Authors:** Qianyu Cheng, Chang Liu, Qiuman Chen, Wenping Luo, Tong-Chuan He, Deqin Yang

**Affiliations:** ^1^ Chongqing Key Laboratory of Oral Diseases and Biomedical Sciences, Chongqing, China; ^2^ Chongqing Municipal Key Laboratory of Oral Biomedical Engineering of Higher Education, Chongqing, China; ^3^ College of Stomatology, Chongqing Medical University, Chongqing, China; ^4^ Department of Endodontics, Stomatological Hospital of Chongqing Medical University, Chongqing, China; ^5^ Department of Stomatology, Hainan Women and Children’s Medical Center, Haikou, China; ^6^ Laboratory Animal Center, Southwest University, Chongqing, China; ^7^ Department of Orthopaedic Surgery and Rehabilitation Medicine, Molecular Oncology Laboratory, The University of Chicago Medical Center, Chicago, IL, United States

**Keywords:** stem cells from the apical papilla (SCAP), hTERT, immortalization, BMP9, osteogenic differentiation

## Abstract

Stem cells from the apical papilla (SCAPs) are promising candidates for regenerative endodontic treatment and tissue regeneration in general. However, harvesting enough cells from the limited apical papilla tissue is difficult, and the cells lose their primary phenotype over many passages. To get over these challenges, we immortalized human SCAPs with lentiviruses overexpressing human telomerase reverse transcriptase (hTERT). Human immortalized SCAPs (hiSCAPs) exhibited long-term proliferative activity without tumorigenic potential. Cells also expressed mesenchymal and progenitor biomarkers and exhibited multiple differentiation potentials. Interestingly, hiSCAPs gained a stronger potential for osteogenic differentiation than the primary cells. To further investigate whether hiSCAPs could become prospective seed cells in bone tissue engineering, *in vitro* and *in vivo* studies were performed, and the results indicated that hiSCAPs exhibited strong osteogenic differentiation ability after infection with recombinant adenoviruses expressing BMP9 (AdBMP9). In addition, we revealed that BMP9 could upregulate ALK1 and BMPRII, leading to an increase in phosphorylated Smad1 to induce the osteogenic differentiation of hiSCAPs. These results support the application of hiSCAPs in tissue engineering/regeneration schemes as a stable stem cell source for osteogenic differentiation and biomineralization, which could be further used in stem cell-based clinical therapies.

## Introduction

Stem cells from the apical papilla (SCAPs), which reside in the apical portion of the epithelial diaphragm, have been confirmed as essential dental-derived mesenchymal stem cells (MSCs) involved in tooth root development and apexogenesis (Sonoyama et al., 2006; Sonoyama et al., 2008; Huang et al., 2009; Wang et al., 2012). Dental MSCs possess characteristics of self-renewal potential, high proliferative ability, and low immunogenicity and can transform into a specialized cell types, such as osteogenic, neurogenic, adipogenic, and chondrogenic cells that display the morphology and biological functions of the mature cells under appropriate induction condition (Maxim et al., 2015). Cells share a specific cell surface marker spectrum (STRO-1^+^, CD146^+^, CD73^+^, CD29^+^, CD90^+^, CD105^+^, and CD34^−^) and can express a variety of osteogenic markers (binding sialoprotein (BSP), alkaline phosphatase (ALP), RUNX family transcription factor 2 (RUNX2), matrix extracellular phosphoglycoprotein (MEPE), etcetera) (Cui et al., 2018; Spagnuolo et al., 2018). These characteristics of dental-derived MSCs are distinct from those of other MSCs, and these cells have been used to treat not only dental diseases but also non-dental diseases such as neurodegenerative lesions, autoimmune diseases, and orthopedic disorders (Yamada et al., 2019). Owing to from immature tissues, SCAPs are rich in early stem/progenitor cells and could be an ideal candidate for regenerative endodontic treatment and tissue regeneration in general. However, harvesting enough cells from the limited apical papilla tissue is difficult, and cells lose their primary phenotype over many passages.

Immortalization has been reported to be an effective method for overcoming these challenges. Cell immortalization can be achieved through human telomerase reverse transcriptase (hTERT) overexpression (Harrington et al., 1997a; Avilion et al., 1996; Harrington et al., 1997b). Telomeres lying in the chromosomes’ distal ends comprise tandem repeats of the TTAGGG sequence (Greider, 1996). Cell division shortens telomere length, and the process stops when these stem cells reach the mortality stage I (M1), the inception when cells cannot proliferate further (Watson, 1972; Lustig, 1999). Telomere length is regulated by a cellular ribonucleoprotein complex called telomerase. It is composed of human telomerase RNA (hTR) (as telomeric template), momentous human telomerase reverse transcriptase (hTERT) with conserved reverse transcriptase motifs, and human telomerase-associated protein 1 (hTEP1) (which is involved in coordinating the structure of telomerase holoenzyme and regulating telomerase regulatory factors) (Bodnar et al., 1998; Counter et al., 1998). Among the constituents, hTERT is most important due to its role in the immortalization process and long-term tumor growth (Ikbale el et al., 2016; Inada et al., 2019). Transcription of hTERT into the human somatic cells causes telomere length elongation and *in vitro* replicative life span extension (Bodnar et al., 1998). Immortalization driven by hTERT overexpression has been reported in many cells related to dental tissue, including periodontal ligament cells derived from deciduous teeth, dental pulp stem cells, and deciduous tooth-derived dental pulp cells (Kitagawa et al., 2007; Hasegawa et al., 2010; Ikbale el et al., 2016; Inada et al., 2019). The establishment of these immortalized cells contributes to studies on cellular mechanisms and regenerative processes in the dental field. However, research on the immortalization of human SCAPs, one of the most promising dental MSCs, is still lacking.

In addition, established immortalized dental MSCs can differentiate into osteogenic cell lineages under appropriate induction conditions (Fujii et al., 2006; Hasegawa et al., 2010). SCAPs possesses higher proliferation potential and mineralization capacity, suggesting that immortalized hSCAPs may also become prospective seed cells in bone tissue engineering (Bakopoulou et al., 2011). Numerous growth factors are involved in determining the lineage of dental-derived MSCs, including BMPs (Balic and Thesleff, 2015). Bone morphogenetic protein 9 (BMP9, aka, growth differentiation factor 2 (GDF2)) has been demonstrated to be one of the most promising inducible factors that could promote MSCs’ osteogenic differentiation (Kang et al., 2004; Luther et al., 2011). Previous research has reported that BMP9 could validly promote osteoblastic differentiation of immortalized SCAPs in mice (Wang et al., 2014). However, the effect of BMP9 on osteoblastic differentiation in human SCAPs and human immortalized SCAPs (hiSCAPs) is yet to be investigated. In addition, BMP9 transduces signals through two transmembrane kinase receptors, known as type I BMP receptors, composed of receptor-like kinase 1 (ALK1) and ALK2, as well as type II BMP receptors, mainly BMP receptor type II (BMPRII) and activin receptor type II B (ActR-IIB), to form an active ligand receptor complex (Tang et al., 2020). Upon this complex formation, BMP promotes the phosphorylation of downstream Smads. BMP9 mainly activates Smad1/5/8 and could activate Smad2 or Smad3 under certain conditions (Star et al., 2010; González-Núñez et al., 2013). There have been studies on BMP9 signaling pathway; however, the underlying molecular mechanism of BMP9-induced osteogenic differentiation of stem cells was rarely reported.

In this study, we established hiSCAPs from extracted immature premolars. We evaluated whether these cell lines retained SCAPs properties and revealed osteogenic potential *in vitro* and *in vivo* upon BMP9 stimulation. The results demonstrated the properties of established hiSCAPs regarding their morphology, proliferation rate, tumorigenic potential, and multipotential differentiation properties compared to those of their parental cells. In addition, we confirmed that BMP9 effectively induced ALP activity, promoted matrix mineralization *in vitro*, and improved the expression level of osteogenic markers. *In vivo* implantation of hiSCAPs infected with AdBMP9 induced robust ectopic bone formation. Furthermore, hiSCAPs overexpression BMP9 could promote BMPRII/Smad and ALK1/Smad activity. Taken together, we reported the establishment of hiSCAP lines with parental cell characteristics and confirmed that these cells may become prospective seed cells in bone tissue engineering, especially when stimulated by BMP9.

## Materials and methods

### Subjects and cell culture

Human developing premolars were collected under approved guidelines from three individuals aged 11–13 years who were clinically healthy and required orthodontic extraction. SCAPs were isolated and cultured according to recognized protocols. The tooth root apical papilla was harvested from the extracted premolar, cleaned with phosphate-buffered saline (PBS, Hyclone, New York, United States), containing 2% penicillin/streptomycin solution (Hyclone, New York, United States), and then treated to 30 min of enzymatic digestion using 3 mg/mL type I collagenase (Sigma, United States). The dispersed cells were suspended in α-MEM (Hyclone, New York, United States) supplemented with 10% fetal bovine serum (FBS, Hyclone, New York, United States) and 1% penicillin/streptomycin after centrifugation. All cultures were maintained in a humidified conditions containing 5% CO_2_ at 37°C.

### Flow cytometric analysis

The expression of MSC-associated surface markers was tested using the third passage of primary cells to analyze the stem cell nature of the hSCAPs. Cells were collected with 0.25% trypsin, washed with sterile PBS, and incubated using specific antibodies against CD29, CD90, or CD45 (Sino Biological, China) at 4°C for 30 min. Samples were tested using a BD Accuri™ C6 flow cytometer (BD Biosciences), and data were coped with Cell Quest software (Becton Dickinson).

### Immortalization of primary hSCAPs

**FIGURE 1:**
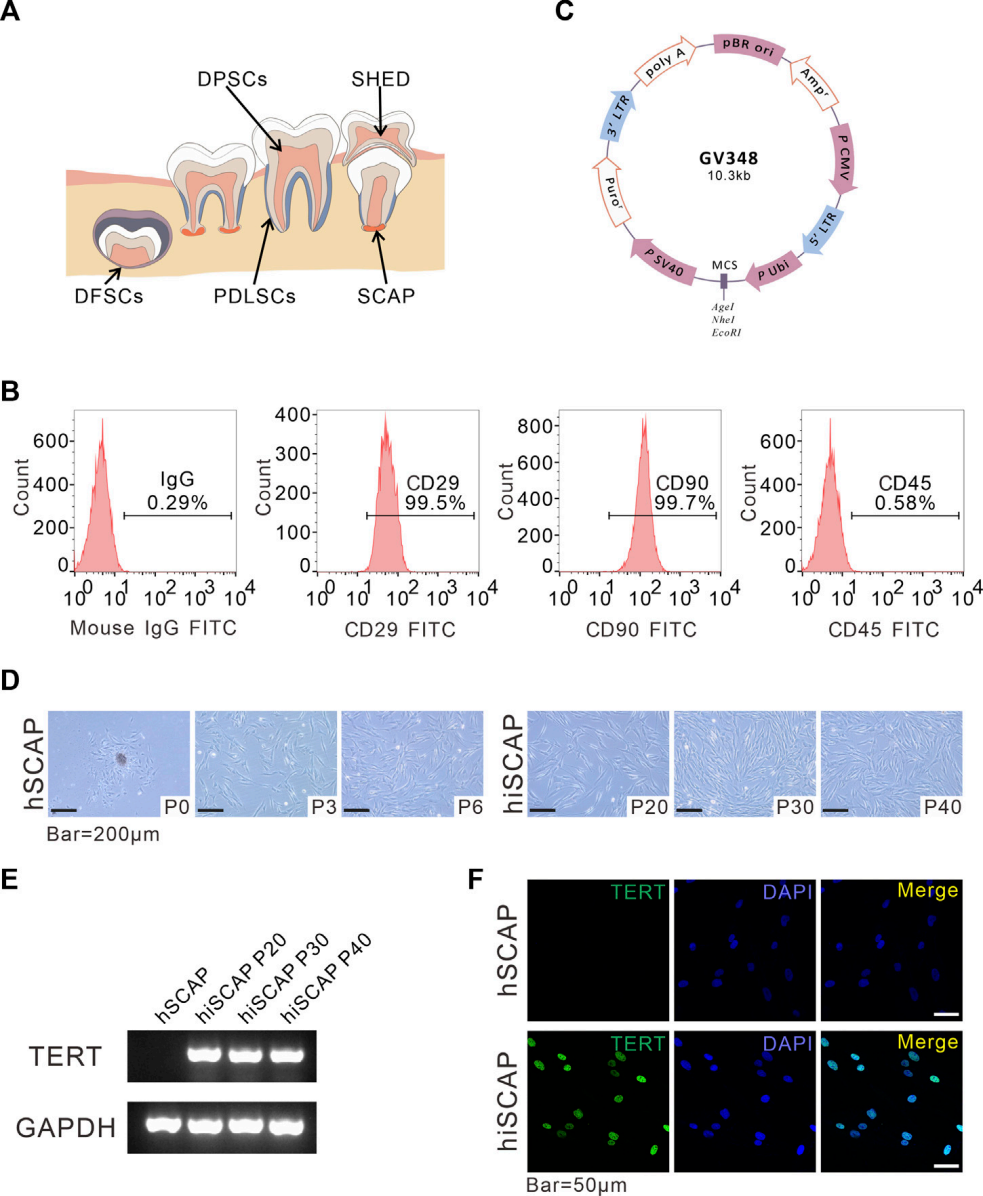
Generation and identification of hiSCAPs. **(A)** A schema graphs of dental derived stem cells. **(B)** Surface markers as assessed by flow cytometry. The obtained hSCAPs were strongly positive for mesenchymal stem cell markers CD29, CD90 and negative for hematopoietic precursor cell marker CD45. **(C)** Structural pattern of plasmid for the stable cell line. **(D)** Morphology of primary hSCAPs and the immortalized cells. Bar = 200 µm. **(E)** The expression of the transgene examined by semi-quantitative PCR. The agarose gels electrophoresis result indicated that TERT was only continuously and stably expressed in hiSCAPs. **(F)** The TERT protein expression in hSCAPs and hiSCAPs as detected by immunofluorescence. The staining (green) results revealed that TERT protein was only expressed in the nucleus of hiSCAPs. Nuclei were counterstained with DAPI. Scale bar = 50 µm.

To establish immortalized hSCAPs, primary cells from three individuals at P1 were mixed, cultured in flasks and then infected with lentiviruses overexpressing hTERT (NM_198,253, Shanghai Genechem Co., Ltd., China). The plasmid profile is presented in Figure 1C. The stable hiSCAPs line were further established by selecting the infected cells with purinomycin for a week. All further experiments were performed using these hiSCAPs and mixed hSCAPs.

### Semi-quantitative polymerase chain reaction (PCR) and quantitative real-time PCR (qPCR)

Total RNA was prepared using Trizol reagent (Takara, Japan) according to the manufacturer’s instructions, and complementary DNA (cDNA) was synthesized using the Reverse Transcription Kit (Takara, Japan). Semi-quantitative PCR was carried out with SYBR Green PCR Master Mix (Takara, Japan). These PCR products containing fluorescent DNA loading buffer (Beyotime, D0076, Jiangsu, China) were separated by agarose gel electrophoresis and photographed under ultraviolet excitation. qPCR was performed using the ABI Prism 7500 Real-Time PCR System (Applied Biosystems) in the volume of 20 μL, which consist of SYBR Green PCR Master Mix (10 μL), PCR primer (2 μL, Sangon Biotech, China) and diluted cDNA (8 μL). Glyceraldehyde 3-phosphate dehydrogenase (GAPDH) was used to normalize the amounts of cDNA in all samples. Specific primer sets used in this study are listed in Table 1.

**TABLE 1:** Primer sequence.

● GAPDH	● COL2A1
Forward: CCA CTC CTC CAC CTT TGA	Forward: CAT CCC ACC CTC TCA CAG TT
Reverse: CAC CAC CCT GTT GCT GTA	Reverse:GGG CAT TTG ACT CAC ACC AG
● TERT	● OPN
Forward: TCA CGG AGA CCA CGT TTC AAA	Forward: ATG ATG GCC GAG GTG ATA GT
Reverse: TTC AAG TGC TGT CTG ATT CCA AT	Reverse: ACC ATT CAA CTC CTC GCT TT
● ALP	● OCN
Forward: GAC CTC CTC GGA AGA CAC TC	Forward: AGC AAA GGT GCA GCC TTT GT
Reverse: TGA AGG GCT TCT TGT CTG TG	Reverse: GCG CCT GGG TCT CTT CAC T
● RUNX2	● ID1
Forward: TGG TTA CTG TCA TGG CGG GTA	Forward: CTA CGA CAT GAA CGG CTG TTA
Reverse: TCT CAG ATC GTT GAA CCT TGC TA	Reverse: CAA CTG AAG GTC CCT GAT GTA G
● LPL	● ALK1
Forward: GAC TCG TTC TCA GAT GCC CTA	Forward: CCT AGC TCA GAT GAT GCG GG
Reverse: ATG GGA TGT TCT CAC TCT CGG	Reverse: GGC TTC TCT GGA CTG TTG CT
● PPARγ	● BMPR2
Forward: CGC CGT GGC CGC AGA TTT GA	Forward: ATC CAG ATT ATT CTT CCT CCT C
Reverse: AGT TGG TGG GCC AGA ATG GCA	Reverse: TCA CGA TGC TGT CAG TAT G
● SOX9	
Forward: CGA AAT CAA CGA GAA ACT GGA C	
Reverse: ATT TAG CAC ACT GAT CAC ACG	

### Immunofluorescence staining

Cells were subjected to 4% paraformaldehyde for 15 min, permeabilized with 0.2% Triton-X100 for 15 min, and then blocked with 10% goat serum for 1 h. Samples were incubated with the primary antibodies listed in Table 2 overnight at 4°C. Alexa Fluor^®^ 488-AffiniPure goat anti-rabbit/mouse IgG (H + L) were used as the secondary antibodies (green). Finally, DAPI (blue) was adopted to counterstain the cells. IgG at an equivalent concentration was used as an isotype control. Images were captured using a laser scanning confocal microscope (LSCM; Leica).

**TABLE 2:** Primary antibodies for Western blotting and IF.

Protein	Specificity	Company	Product code
TERT	Rabbit	Abcam	ab230527
CD44	Mouse	Abcam	ab6124
C-KIT	Rabbit	Bioss	bs-10005R
NESTIN	Rabbit	Bioss	bs-0008R
VIMENTIN	Rabbit	Abcam	ab92547
STRO-1	Mouse	Santa Cruz	sc-47733
CD166	Rabbit	Bioss	bs-1251R
KI67	Rabbit	Abcam	ab15580
CD34	Rabbit	Abcam	ab81289
GAPDH	Mouse	Abcam	ab8245
ALP	Rabbit	Abcam	ab229126
PPARγ	Rabbit	Cell Signaling Technology	#2435
SOX9	Rabbit	Abcam	ab185966
ID1	Mouse	Santa Cruz	sc-133104
ALK1	Rabbit	Novus Biologicals	NBP1-90254
BMPR2	Rabbit	Abcam	ab96826
P-SMAD1	Rabbit	Abcam	ab214423
T-SMAD1	Rabbit	Abcam	ab33902

### Proliferation assay

SCAPs and hiSCAPs were plated into 96-well culture plates at a concentration of 1*10^3^/well and cultured under conventional conditions to evaluate the cell proliferation rate of hSCAPs and hiSCAPs. The CCK-8 assay was carried out using Cell Counting Kit protocol (CCK-8; Bioss, Beijing, China) for 7 consecutive days. Reaction products were measured spectrophotometrically at a wavelength of 450 nm.

### Crystal violet staining

SCAPs and hiSCAPs were seeded into 35 mm cell culture dish with the same initial concentration. Cell samples were fixed in 4% paraformaldehyde for 15 min and then stained with crystal violet for 10 min at each indicated time point. Images of macrographic staining were captured. Cells were dissolved in 10% acetic acid for 20 min with agitation, and the absorbance value was measured at 590 nm for further quantitative analysis.

### Cytogenetic analysis

Metaphase spreads were prepared from exponentially growing hSCAPs and hiSCAPs according to standard protocols (Vorsanova et al., 2010). Cells were tested using standard Giemsa staining. Fifteen metaphase cells were karyotyped for both cell types using the CytoVision system (Leica Biosystems, Germany). Chromosome identification was carried out following the International System for Chromosome Nomenclature.

### *In vivo* tumorigenic assay

Cells labeled with firefly luciferase were harvested with trypsin, suspended in 60–80 uL PBS, and kept on ice until injection into the host mice (Westerman and Leboulch, 1996; Li et al., 2013). Prior to injection, the mice were anesthetized using isoflurane gas. Six-week male mice were injected subcutaneously in the left flank with hiSCAPs (1 × 107/group) and in the right flank with the human squamous carcinoma cell SCC-15 (as positive control). Tumorigenic potential of hiSCAPs was assessed using a Xenogen IVIS 200 imaging system (Luo et al., 2008; Li et al., 2014; Liao et al., 2015). The time when the fluorescence signal disappeared was recorded. Xenogen bioluminescence images on day 2 and 14 are presented.

### Multilineage differentiation assays

Cells were planked into plates containing basal complete medium and cultured to 80%–90% confluency. The basal medium was then replaced with the corresponding differentiation-inducing mixture. The medium was replaced with a DMEM with low glucose (Hyclone, New York, United States) complete medium containing 10 nM dexamethasone (Sigma, United States), 50 mg/mL ascorbic acid 2-phosphate (Sigma, United States), and 10 mM β-glycerophosphate (Sigma, United States) to induce osteogenic differentiation. Adipogenic differentiation induction medium contained 100 nM dexamethasone (Sigma, United States), 50 μg/mL ascorbic acid 3-phosphate (Sigma, United States), and 50 μg/mL indomethacin (Sigma, United States). Chondrogenic differentiation was induced using a chondrogenic differentiation medium (Lonza, Basel, Switzerland) supplemented with recombinant TGFb3 protein (R&D Systems, Minneapolis, MN, United States) (Li et al., 2020).

### Western blot analysis

Samples were lysed in radioimmunoprecipitation assay (RIPA) buffer (Beyotime, Jiangsu, China), and protein concentrations were assessed using the BCA Protein Assay Kit (Beyotime, Jiangsu, China). Protein lysates (25 µg/lane) were separated by SDS-PAGE and then transferred to a nitrocellulose membrane (Millipore, Billerica, MA, United States). Membranes were incubated with primary antibodies listed in Table 2 and corresponding horseradish peroxidase-conjugated goat anti-rabbit/mouse secondary antibodies (1:2000; Zhongshan Golden Bridge, China). Blots were visualized using a chemiluminescence kit (Beyotime, Jiangsu, China). The ImageJ software was adopted to quantify the bands.

### Generation and amplification of recombinant adenoviruses expressing BMP9 and GFP

Recombinant adenoviruses expressing BMP9 were generated using AdEasy technology (Luo et al., 2007). The coding region of human BMP9 was PCR-amplified, cloned into the adenoviral shuttle vector, and then these vectors were used to generate and amplify adenovirus in HEK-293 cells. A similar adenovirus expressing only GFP (AdGFP) served as controls (Luo et al., 2004; Tang et al., 2009). We used 5 μg/mL polybrene (Solarbio, China) to enhance adenoviral infection efficiency (Zhao et al., 2014).

### ALP staining and ALP activity assay

SCAPs and hiSCAPs were seeded in 24-well plates with the same cell density and further infected with AdBMP9 or AdGFP. ALP staining and activity assays were performed following the guidance of the NBT/BCIP staining kit and ALP Assay Kit instructions (Beyotime, Shanghai, China).

### Alizarin red S mineralization staining and quantification

After 3 weeks of osteogenic differentiation induction, cells were stained with 1% alizarin red S (ARS) solution (pH 4.0–6.0, Sigma, United States) for 15 min at RT. The calcium mineral deposits were stained red and recorded using a microscope. For the quantitative analysis, the deposits were dissolved in 10% cetylpyridinium chloride monohydrate (Solarbio, China) at RT for 1 h. The dissolved products were measured spectrophotometrically at a wavelength of 405 nm. All experiments were performed in triplicate.

### Cell implantation, ectopic bone formation, and micro-CT analysis

Stem cell-mediated ectopic bone formation was carried out according to established protocols (Kang et al., 2004). Briefly, SCAPs and hiSCAPs (1 × 10^7^/group) infected with AdBMP9 or AdGFP for 16 h were subcutaneously injected into the flanks of BALB/c nude mice. Eight weeks after surgery, mice were killed by CO2 overdose, and the implantation sites were harvested and fixed in 4% paraformaldehyde. Samples were scanned using μCT (Viva CT 40, Scanco Medical, Bassersdorf, Switzerland) and then reconstructed in three-dimension. The results were quantitatively analyzed with μCT V6.1 software (Wang et al., 2014).

### Histological evaluation

The scanned samples were re-fixed, decalcified, dehydrated, and embedded in paraffin. Serial sections from each group were stained with hematoxylin and eosin (H&E). Trichrome staining of corresponding sections was performed with Masson’s Trichrome Stain Kit (Solarbio, China) following the manufacturer’s protocol.

### Statistical analysis

The experiment was performed independently three times. Data were expressed as the mean ± SD. All statistical analyses were performed using SPSS Statistics version 20.0 (IBM Corp.). Student’s t-test was conducted to determine whether there were significant differences between the two sets of data. For comparisons between multiple groups, One-way analyses of variance (ANOVA) were performed to detect the significant effects of the variables and Tukey’s multiple comparison test was used to compare the means of each of the groups. Asterisks indicate the degree of statistical differences: *p < 0.05, **p < 0.01, ***p < 0.001.

## Results

### Generation and identification of hiSCAPs

Dental tissue is rich in stem cells, such as periodontal ligament stem cells (PDLSCs), dental pulp stem cells (DPSCs), dental follicle stem cells (DFSCs), SCAPs, and stem cells from human exfoliated deciduous teeth (SHED), all of which have self-renewal potential, high proliferative ability, and low immunogenicity, and multipotential differentiation properties (Figure 1A). SCAPs are dental MSCs derived from soft tissue in the apical portion of developing root (Figure 1A). Flow cytometric assay was performed using MSC markers, including CD29, CD90, and the MSC-negative marker CD45 to characterize the obtained SCAPs. These cells were strongly positive for CD29 (99.5%) and CD90 (99.5%), whereas negative for the leukocyte precursor marker CD45, which indicated the stromal origin without hematopoietic precursor contamination (Figure 1B).

To establish immortalized human SCAPs, cells were infected with lentiviruses overexpressing hTERT and selected with purinomycin for a week. The plasmid profile is illustrated in Figure 1C. We then compared the morphology of cells with and without immortalization. After the initiation of primary culture, small, round, spindle-shaped cells were observed migrating from the attached apical papilla fragments. The cells spread along the surface of the culture flasks and exhibited a typical fibroblast-like morphology. Immortalized cells were also spindle-shaped, and there were no obvious morphological differences between hSCAPs and hiSCAPs (Figure 1D). Next, we tested the mRNA expression of hTERT in these two cell lines via semi-quantitative PCR, and the results revealed that hTERT was only continuously and stably expressed in hiSCAPs (Figure 1E). This result was consistent with the immunofluorescence staining; that is, the TERT protein was only expressed in the nucleus of hiSCAPs, and the expression remained stable even in the 40th passage of hiSCAPs (Figure 1F).

### Characteristics of hiSCAPs

#### Cell proliferation and tumorigenic potential

**FIGURE 2:**
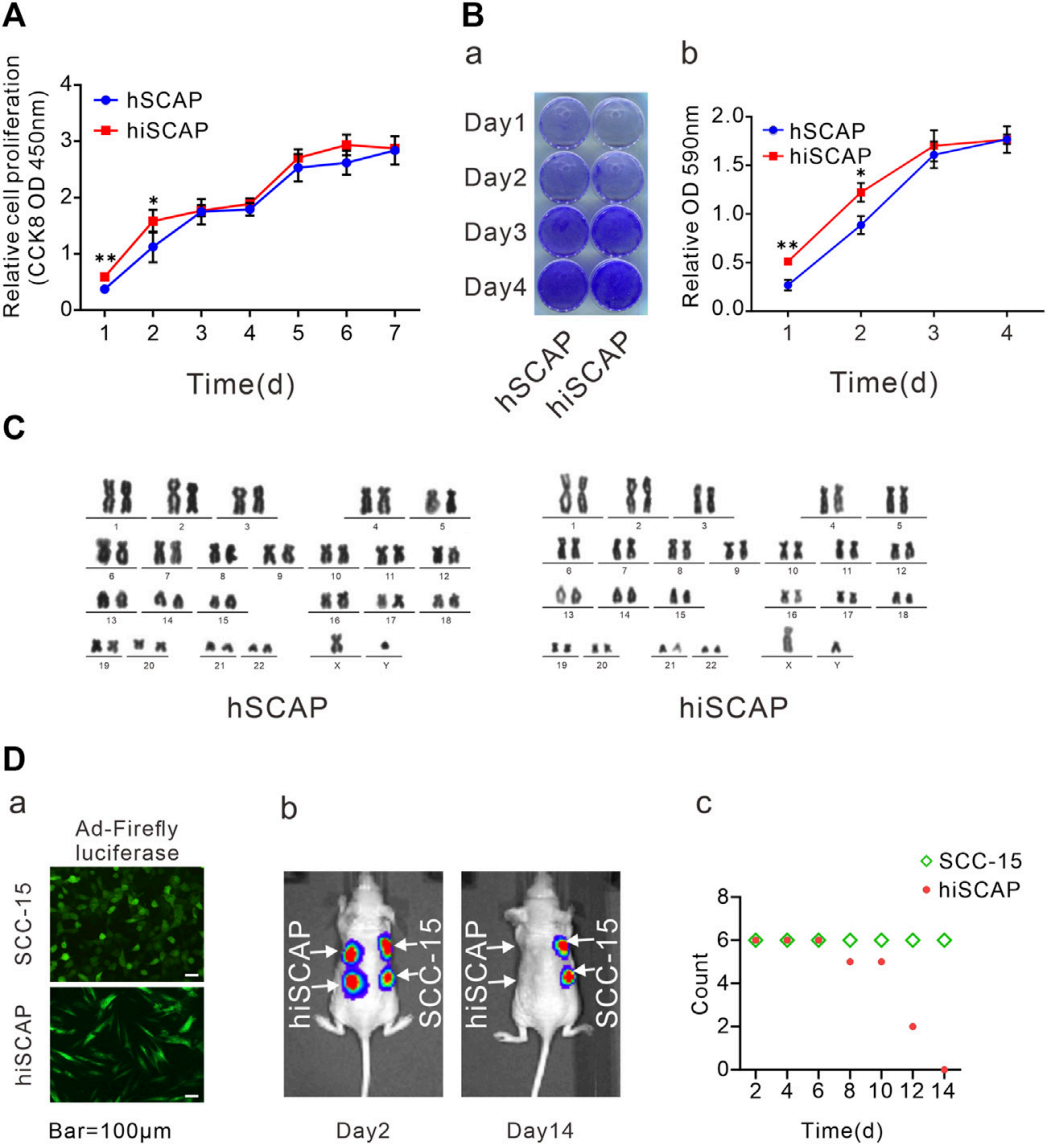
Cell proliferation and tumorigenic potential. **(A)** The proliferation rate of hSCAPs and hiSCAPs detected by CCK-8 assay. SCAPs (P4) and hiSCAPs (P28) were seeded at a low density and tested at the indicated time scale. **(B)** Crystal violet staining. Two cells were seeded at 30 mm dish with similar initial density and then fixed with 4% paraformaldehyde for crystal violet staining. The stained cells were dissolved in 10% acetic acid and measured the absorbance value at 590 nm. **(C)** HiSCAPs revealed no abnormality of karyotype. **(D)** Analysis of the tumorigenic risk *in vivo*. (a) HiSCAPs and SCC-15 expressing the firefly luciferase were injected subcutaneously to both flanks of the back on nude mice. Scale bar = 100 µm. (b) Bioluminescence imaging on day 2 and 14. (c) The hiSCAPs signals disappeared completely within 14 days, while the SCC-15 signals remained detectable. All values were the mean ± SDs; *, *p* < 0.05 and **, *p* < 0.01.

The proliferation potential was detected by CCK-8 cell proliferation assays and crystal violet staining to determine whether immortalization affects the proliferation ability of SCAPs. CCK-8 assays demonstrated that immortalized cells exhibited slightly increased proliferation in the first 2 days, whereas they had similar proliferative potential as the primary cells after culturing for 3 days (Figure 2A). Similarly, crystal violet staining confirmed that hiSCAPs proliferated slightly faster only in the early stages (Figure 2B). Significantly, hiSCAPs could be passaged more than 40 times and revealed stable proliferative potential even beyond 4 months.

HiSCAPs may display chromosomal changes after multiple passages owing to genomic instability; thus, karyotype analysis was performed. G-banding of metaphase chromosomal spreads from hiSCAPs revealed a normal 46, XY male chromosomal complement with no polyploid mutation or chromosomal deletion, similar to the hSCAPs (Figure 2C). We also examined the *in vivo* tumorigenesis of the immortalized cells. SCC-15, a human squamous carcinoma cell line, was used as the positive control. HiSCAPs and SCC-15 were labeled with firefly luciferase and subsequently injected subcutaneously into the nude mice (Figure 2D-a). The animals underwent whole-body live bioluminescence imaging from the second day after treatment. On day 2, both bioluminescence signals were detectable. The hiSCAPs signals gradually decreased and became undetectable on day 14, whereas the SCC-15 signals persisted (Figure 2D-b). The time at which bioluminescence signals disappeared was also recorded. The result revealed that the hiSCAPs signals disappeared completely within 14 days, while the SCC-15 signals remained detectable (Figure 2D-c). In addition, we monitored immunodeficient mice until the SCC-15 injection group formed evident tumor transformation. None of the hiSCAPs led to tumorigenesis, indicating that hiSCAPs were non-tumorigenic despite their long-term proliferative potential.

#### Stemness and multipotency

**FIGURE 3:**
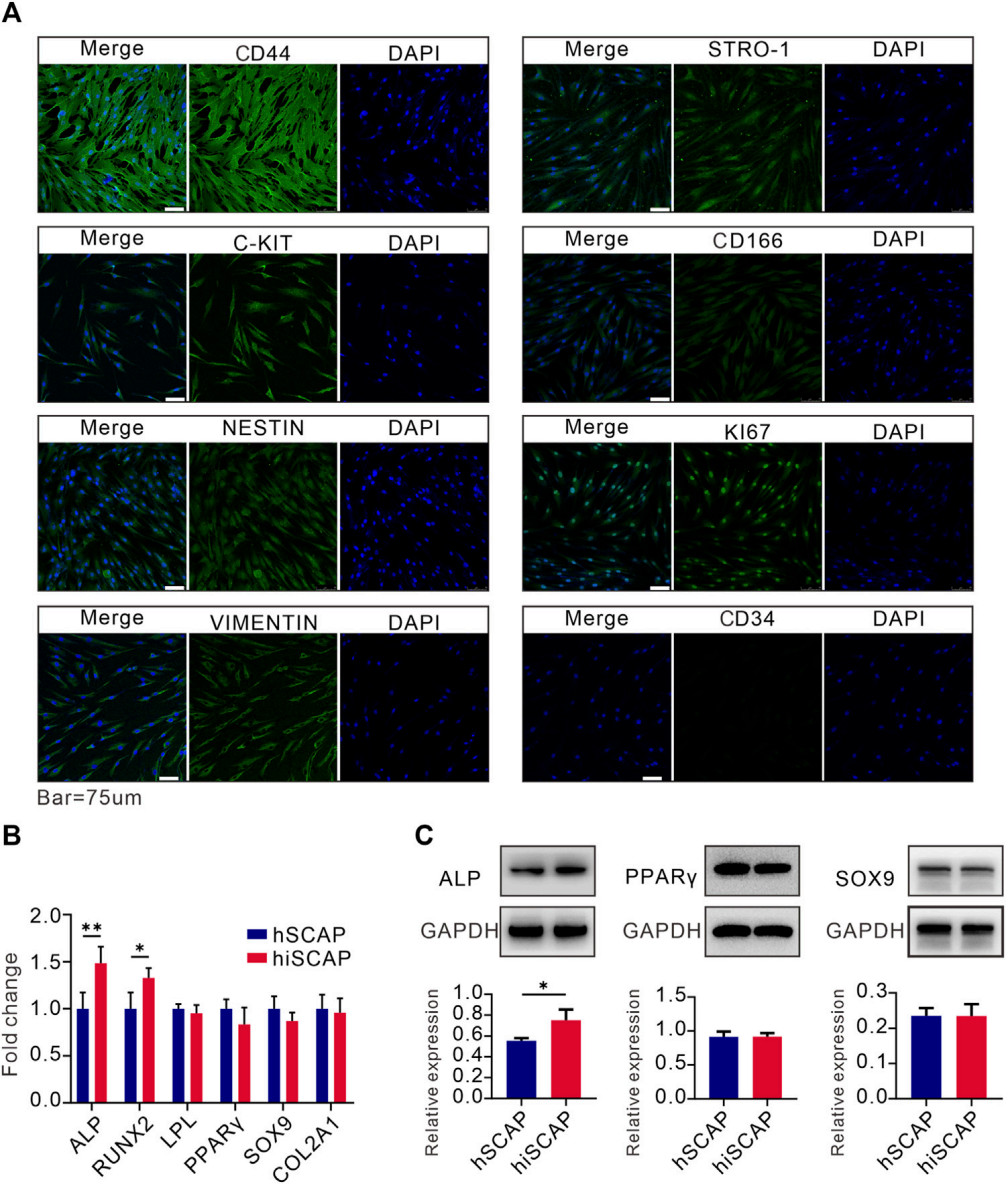
Stemness and multipotency. **(A)** The expression of mesenchymal and progenitor markers in hiSCAPs. IgG at an equivalent concentration was used as an isotype control (not shown). **(B, C)** The mRNA and protein expression of osteogenic, adipogenic, and chondrogenic differentiation markers. Target gene and protein expression levels were normalized to the corresponding GAPDH levels. Each assay condition was performed in triplicate. Quantitative analysis of Western blot was analysed using QuantityOne software. All values were the mean ± SDs; *, *p* < 0.05 and **, *p* < 0.01.

Moreover, immunofluorescence staining was conducted to examine the expression of mesenchymal and progenitor biomarkers in hiSCAPs. Cells were positive for KI67, C-Kit, CD44, VIMENTIN, CD166, STRO-1, and NESTIN, but negative for CD34 expression (Figure 3A). These results indicated that hiSCAPs exhibit characteristics of MSCs.

We then tested the multilineage differentiation potential of hiSCAPs using different differentiation induction assays. Real-time PCR analysis demonstrated that hSCAPs and hiSCAPs had similar mRNA expression level of adipogenic (LPL and PPAR-γ) and chondrogenic (SOX9 and COL2A1) differentiation markers. Nevertheless, the osteogenic differentiation markers (ALP and RUNX2) expression was higher in hiSCAPs than in hSCAPs (Figure 3B). Western blot was performed to detect protein expression in hSCAPs and hiSCAPs. The results confirmed that the protein expression of PPAR-γ and SOX9 in both cell types was similar, whereas ALP was higher in hiSCAPs (Figure 3C). These results implied that hiSCAPs may reveal a stronger osteogenic differentiation capacity than primary cells after induction.

### BMP9 effectively induced osteogenic activity of hSCAPs and hiSCAPs

**FIGURE 4:**
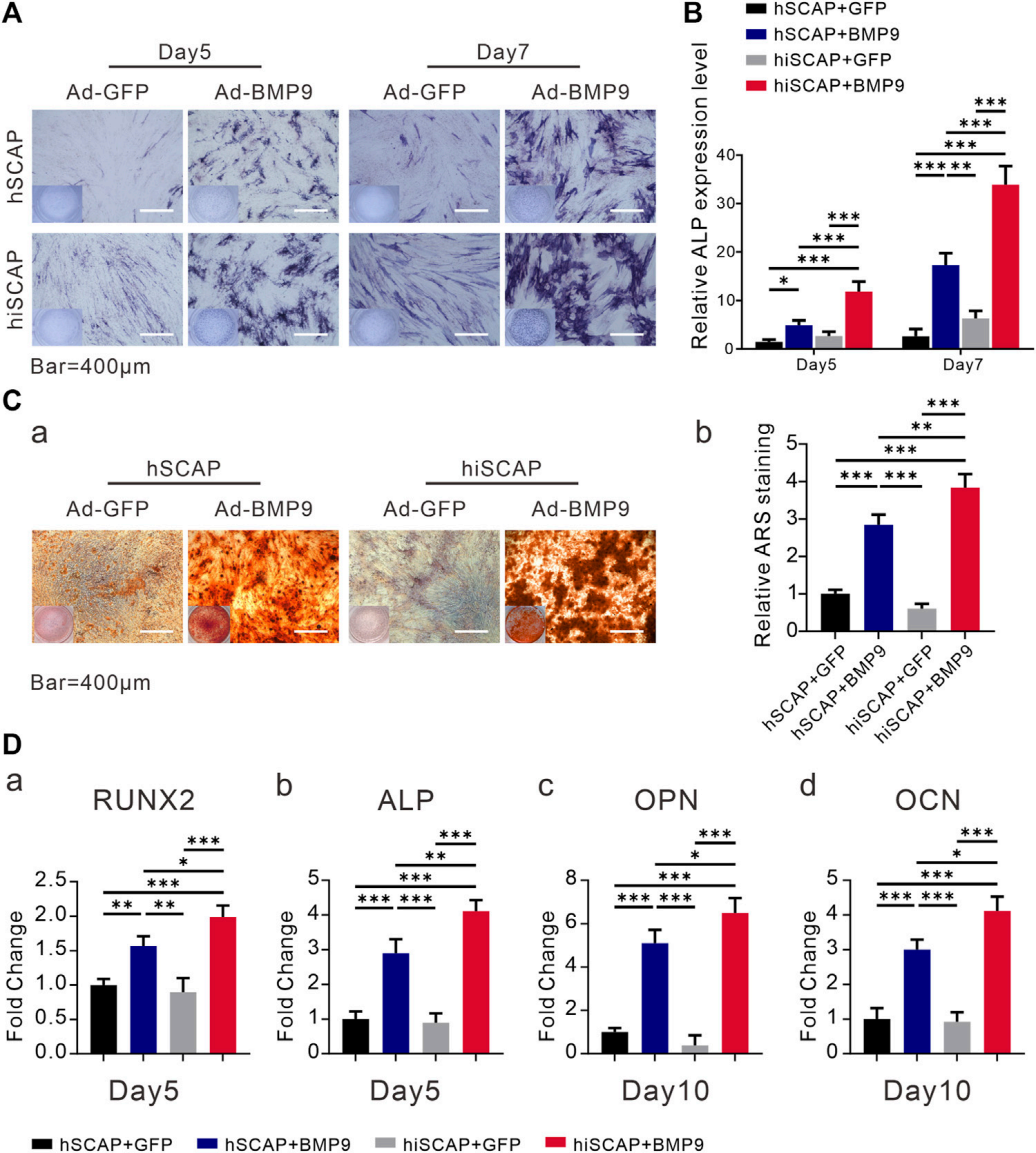
BMP9 effectively induced osteogenic activity of hSCAPs and hiSCAPs. **(A)** ALP staining. **(B)** ALP activity assay. Cells were seeded in 24-well culture plate and infected with AdBMP9 or AdGFP (as control). ALP staining and activity were observed on days 5 and 7. Scale bar = 400 µm. **(C)** Alizarin red S staining and quantification. Two cells were cultured in matrix mineralization medium for 21 days. (a) Matrix mineralization nodules were stained with 1% alizarin red S solution. (b) Stained areas were extracted with 10% cetylpyridinium chloride solution for quantification analysis. **(D)** The mRNA expression levels of osteogenic genes as determined by qPCR on days 5 and 10. All experiments were performed in triplicate. Values were the mean ± SD; *, *p* < 0.05, **, *p* < 0.01, and ***, *p* < 0.001.

In order to further investigate whether hiSCAPs could become prospective seed cells in bone tissue engineering, *in vitro* studies were performed. BMP9 is reported to be able to induce the osteogenic differentiation of stem cells. We infected hSCAPs and hiSCAPs with AdBMP9 to determine whether these cells revealed osteogenic differentiation capacity. ALP staining and activity assays were performed on day 5 and 7, respectively. The staining result suggested that BMP9 could effectively induce ALP activity in both cells, and this effect was BMP9 dose-dependent. More importantly, hiSCAPs stimulated with BMP9 exhibited more intense staining than hSCAPs treated with BMP9 (Figure 4A). Moreover, quantitative ALP assays were performed, which revealed similar results (Figure 4B). We also tested late-stage mineralization in BMP9-induced hiSCAPs and hSCAPs by alizarin red staining and discovered that the staining of both cells was markedly enhanced after infection with AdBMP9, whereas hiSCAPs stimulated by BMP9 formed more calcified deposits. Quantitative analysis confirmed this finding (Figure 4C).

Furthermore, we examined the mRNA expression level of bone-related genes in hSCAP-AdGFP, hSCAP-AdBMP9, hiSCAP-AdGFP, and hiSCAP-AdBMP9. Real-time PCR analysis confirmed that all cells expressed bone-related genes, such as ALP, RUNX2, OPN, and OCN. It was also indicated that the expression of genes involved in osteoblastic differentiation was notably upregulated after infection with Ad-BMP9, and this upregulation was more significant in hiSCAP-AdBMP9 than in hSCAP-AdBMP9 (Figure 4D).

### BMP9 induced ectopic bone formation of hSCAPs and hiSCAPs

**FIGURE 5:**
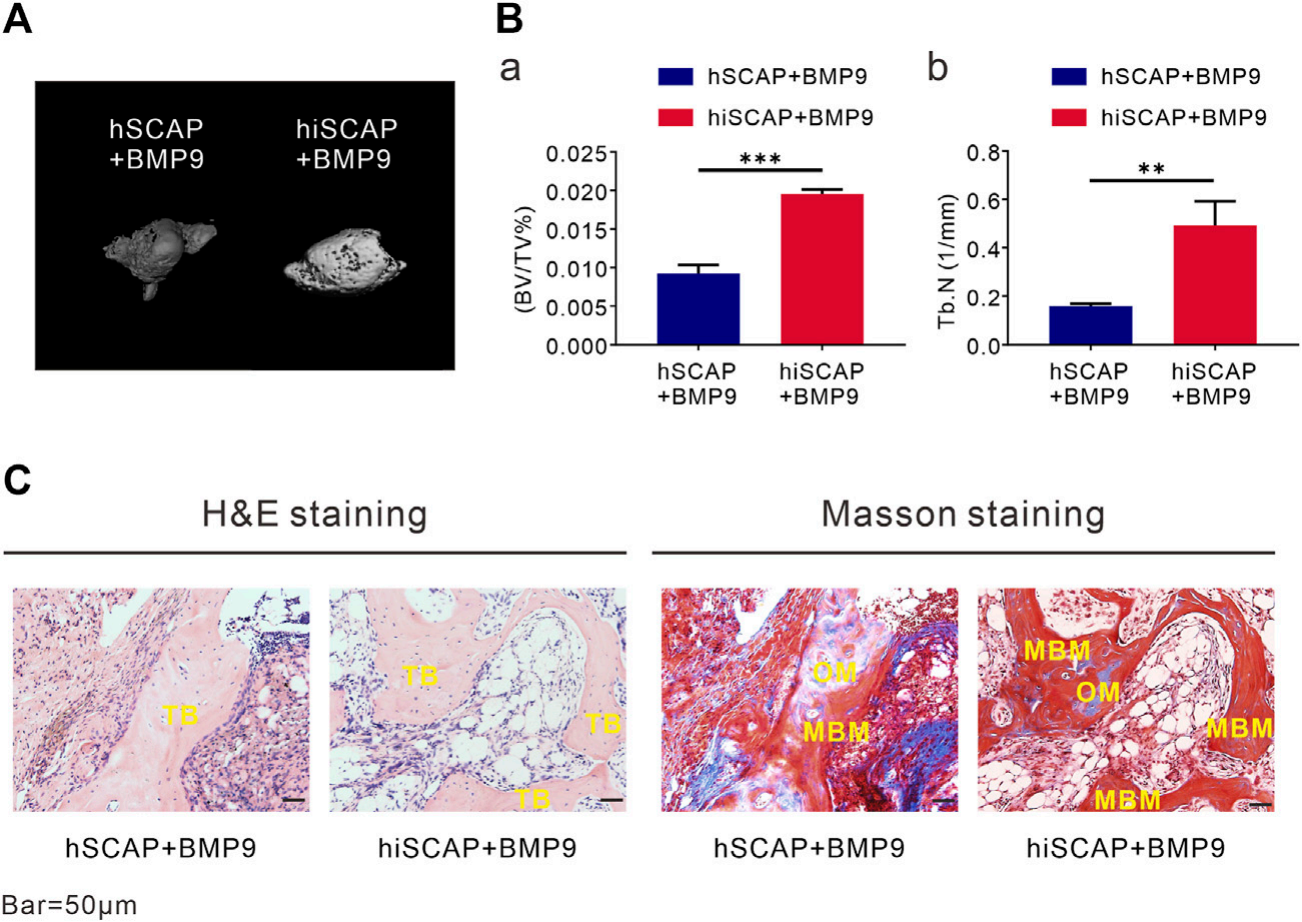
BMP9 effectively induced ectopic bone formation of hSCAPs and hiSCAPs. Cells infected with AdBMP9 or AdGFP were harvested for subcutaneous injections. **(A)** µCT analysis. Eight weeks after surgery, mice were sacrificed by CO2 overdose, and the implantation sites were harvested for 3D volumetric analysis. **(B)** Quantitative analyses of (a) bone volume (BV)/total volume (TV) and (b) trabecular number (Tb.N). **, *P* < 0.01, ***, *p* < 0.001. **(C)** Haematoxylin and eosin (H & E) staining and Trichrome staining. Representative results were shown. TB, trabecular bone; OM, osteoid matrix; MBM, mineralized bone matrix. Scale bar = 50 µm.

To explore the induction effect of BMP9 on the osteogenesis of hiSCAPs *in vivo*, ectopic bone formation was conducted via established cell implantation assay. A sufficient number of infected cells were injected subcutaneously into the flanks of nude mice. Eight weeks after implantation, robust bony masses were harvested from the AdBMP9 infected groups, while masses from hSCAP-AdBMP9 were smaller than those from hiSCAP-AdBMP9. No masses were observed in the AdGFP-infected group. Further, micro-CT imaging revealed a more intuitive general difference (Figure 5A). Quantitative analysis of the volume (bone volume fraction, BV/TV) and quantity (number of trabeculae, Tb.N) of new bone tissue demonstrated that BMP9 could induce *in vivo* bone formation, and more bone had formed in hiSCAP-AdBMP9 than in hSCAP-AdBMP9 (Figure 5B). Moreover, H&E histological evaluation indicated that hSCAP-AdBMP9 and hiSCAP-AdBMP9 formed obvious trabecular structures (Figure 5C). Concurrently, trichrome staining confirmed that hSCAP-AdBMP9 and hiSCAP-AdBMP9 formed mature and mineralized bone matrices, though the samples from hiSCAP-AdBMP9 revealed higher maturity and mineralization (Figure 5C). These *in vivo* results collectively suggested that hiSCAPs could differentiate into osteogenic cell lineages after effective induction.

### BMP9 promoted BMPRII/Smad and ALK1/Smad activity in hiSCAPs

**FIGURE 6:**
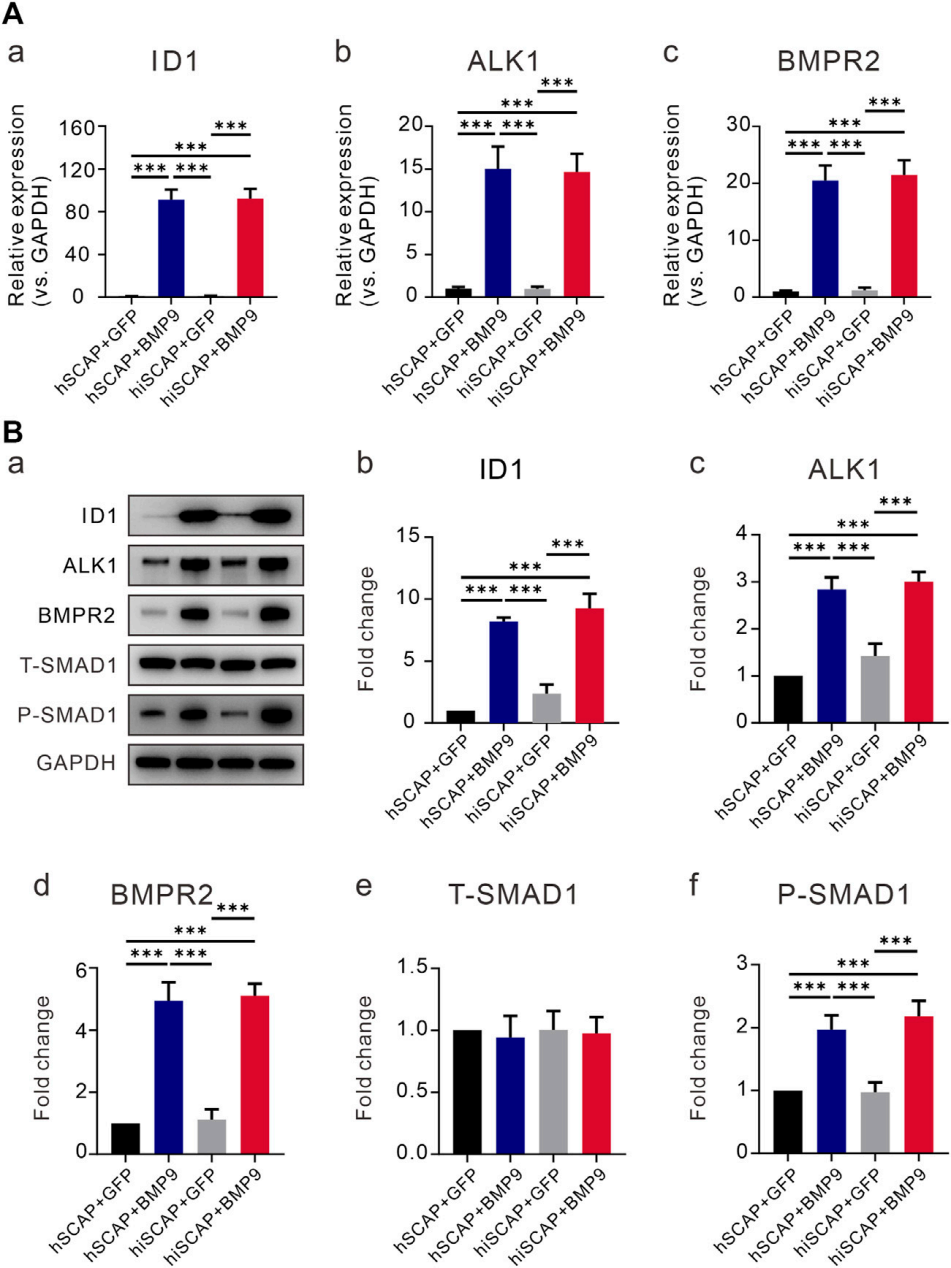
BMP9 promoted BMPR2/Smad and ALK1/Smad activity in hiSCAPs. **(A)** The mRNA expression levels of (a) ID1 and BMP receptors (b) BMPRII and (c) ALK1 in hSCAPs and hiSCAPs. **(B)** The protein expression of (b) ID1, (c) BMPRII, (d) ALK1, (e) total and (f) phosphorylated Smad-1 was analyzed by Western blot. The grey intensity of bands was analyzed using ImageJ software. Each assay condition was performed in triplicate. ***, *p* < 0.001.

We confirmed that BMP9 effectively induced the osteogenic activity of hiSCAPs. However, the signaling mechanisms by which BMP9 promotes the osteogenic differentiation of hiSCAPs remain undefined. We first tested the expression of the target gene inhibitor of DNA binding 1 (ID1). The results demonstrated that the expression of ID1 was upregulated upon infection with AdBMP9 (Figures 6A-a, B-b). Previous studies have confirmed that ALK1 and BMPRII are critical for osteoblast differentiation of MSCs (Luo et al., 2010; Wu et al., 2010). We also proved that BMP9 could increase the expression of the ALK1 and BMPRII in hSCAPs and hiSCAPs both at gene level (Figures 6A-b, A-c) and protein level (Figures 6B-c, B-d). We then tested the activation of canonical Smad-1/5/8 pathways, which are BMP-specific receptor-regulated Smad proteins. The expression of total Smad1 and phosphorylated Smad1 was tested by Western blot, and the results revealed strong expression of phosphorylated Smad-1 in hSCAPs and hiSCAPs treated with BMP9 (Figures 6B-e, 6B-f). These results confirmed that BMP9 could upregulate ALK1 and BMPRII in hSCAPs and hiSCAPs, increasing phosphorylated Smad1 to induce osteogenic differentiation. It was also suggested that hiSCAPs may be a stable cell line for mechanistic research on differentiation and regeneration.

## Discussion

Tooth development comprises the initiation, bud, cap, bell stage, and final maturation (Tucker and Sharpe, 2004). Dental papilla is possibly an appropriate donor for osteo/odontoblast differentiation and could promote the formation and stability of the dental pulp dentin complex. Tip portion of the dental papilla is termed the apical papilla, and it moves apically to promote the continued formation of root dentin and dental pulp (Tziafas and Kodonas, 2010). Significantly, there is a stem/progenitor cell pool lying between the apical papilla and dental pulp, including SCAPs, DPSCs, etcetera, which play an important role in root growth. These cells can be non-invasively isolated from discarded teeth and possess the characteristics of immune privilege status. Cells do not cause an allogeneic reaction and suppress T-cell proliferation, which indicates that cells harvested from a single donor could form a reserve pool *in vitro* for multiple recipients (Le Blanc and Ringdén, 2007). SCAPs own higher population doubling capacity, exhibit stronger proliferation potential, and are more effective for tooth development and regeneration than other dental MSCs (Sonoyama et al., 2006; Bakopoulou et al., 2011). Thus, SCAPs may be an ideal source for many areas of regenerative medicine.

Dental-derived MSCs maintain low grade of senescence even till passage 15, suggesting the safety of transplanting these MSCs for stem cell therapy (Diomede et al., 2017). However, like other stem cells, SCAPs has a limited lifespan *in vitro* and lose their primary phenotype after cultured for a long time *in vitro*. Benzopyrene (BaP), radiation, and ectopic expression of viral oncogenes have been discovered to rescue this phenomenon; however, phenotypic transformation, karyotypic instability, and tumorigenicity in nude mice are the main problems that cannot be ignored (Gudjonsson et al., 2004). Currently, the immortalization of stem cells is an appropriate method for expanding these cells in large quantities without changing their characteristics. In this study, we successfully immortalized hSCAPs with lentiviruses overexpressing hTERT to meet the application needs of hSCAPs. We demonstrated that immortalized cells acquire long-term proliferative activity without tumorigenic potential. Cells were also positive for MSC markers and exhibited multiple differentiation potentials after the differentiation induction. In addition, the results showed that BMP9 effectively induced the osteogenic activity of hSCAPs and hiSCAPs, and the immortalized cells after BMP9 stimulation revealed higher ALP activity and more calcified deposits than the primary cells. Furthermore, ectopic bone formation assay indicated that hSCAPs and hiSCAPs could exhibit *in vivo* osteogenic differentiation ability when infected with AdBMP9, whereas the bone matrices from hiSCAP-AdBmp9 revealed higher maturity and mineralization. Finally, we confirmed that BMP9 could increase the expression of its osteogenic receptors BMPRII and ALK1 in a Smad-dependent manner to induce the osteogenic differentiation of hSCAPs and hiSCAPs.

Immortalization of target cells could be achieved through overexpression of oncogenes and inhibition of tumor suppressor genes (vom Brocke et al., 2006). Several studies have reported that immortalization can be established by transfection with simian virus 40 (SV40) or human papilloma virus 16 (HPV16) (Pirisi et al., 1987; Lechner and Laimins, 1991). Dental-derived MSCs are clonogenic cells with excellent stability and plasticity. Cells revealed no abnormality of karyotype even after 60 population doublings (Avinash et al., 2017; Trubiani et al., 2019). However, overexpression of the SV40 large T antigen may contribute to chromosomal aberrations and polyploidy, while the HPV16 protein could lead to chromosomal instability, such as centrosome abnormalities (Stewart and Bacchetti, 1991; Duensing et al., 2001). Furthermore, ectopic expression of hTERT is also a promising strategy to immortalize cells. Human dental pulp cells (Kamata et al., 2004; Kitagawa et al., 2007; Ikbale el et al., 2016), gingival fibroblasts (Kamata et al., 2004), and periodontal ligament cells (Kamata et al., 2004; Fujii et al., 2006) have been reported to be immortalized by this method. Unfortunately, research on the immortalization and subsequent application of SCAPs has not been conducted. Our study is the first to demonstrate successful immortalization of hSCAPs by overexpressing hTERT.

Human generative cells, cancer cells, and some stem cells have a high TERT activity (Kovalenko et al., 2010; Wang et al., 2013). Research has discovered that hTERT is highly active in undifferentiated DPSCs, while this activity decreases gradually following cell passage (Potdar and Jethmalani, 2015). We tested the expression of hTERT in primary hSCAPs and hiSCAPs. The hTERT gene was continuously and stably expressed in hiSCAPs, whereas TERT expression was almost undetectable in P3 primary hSCAPs. In addition, compared to the original primary cells, the morphology of the immortalized cells was not altered, even after 40 generations. These results suggested that stable cell clones hiSCAPs were formed and that overexpression of the TERT gene extended the lifespan of stem cells.

Seed cells for tissue engineering require proliferation and differentiation potential. The possible occurrence of characteristic alterations after immortalization is a major problem that cannot be ignored. In this research, we examined the proliferation, tumorigenesis, stemness, and multipotential differentiation potential of hiSCAPs in detail, with primary cells as the control. It has been reported that human stromal cells immortalized by SV40T-Ag grow faster than primary stromal cells, whereas the growth rate of hTERT-transfected cells is similar to that of primary cells (Kawano et al., 2003). As demonstrated in this research, hiSCAPs also displayed similar proliferative potential with the primary cells after culturing for 3 days, although exhibited slightly increased proliferation in the first 2 days. Importantly, hiSCAPs could be passaged more than 40 times and revealed stable proliferative potential beyond 4 months, indicating that overexpression of the TERT gene extended the lifespan of stem cells.

In addition, long-term *in vitro* culture of stem cells usually results in impaired differentiation ability (Christiansen et al., 2000). Our results indicated that the 30th passage of immortalized cells were still positive for KI67, C-KIt, CD44, VIMENTIN, CD166, STRO-1, and NESTIN, which indicated that hiSCAPs exhibited MSC characteristics. Moreover, after culturing in various differentiation induction media, the expression of osteogenic, chondrogenic, and adipogenic markers in hSCAPs and hiSCAPs were significantly upregulated, which indicated potential to differentiate with multiple directions of both cells. In addition to dental-derived stem cells, other immortalized cells are also reported to retain the phenotypic characteristics of the parental cells, including human osteoblastic cells, adipose-derived stem cells, and corneal epithelial cells (Wang et al., 2013; Kim et al., 2016; Pérez-Campo et al., 2017). Interestingly, after osteogenesis induction, the expression of osteogenic markers ALP and RUNX2 was higher in hiSCAPs than in hSCAPs. This is also consistent with previous reports that MSCs overexpressing TERT reveal increased osteogenic potential, while a lack of telomerase could affect the differentiation of hMSCs (Liu et al., 2004). These results demonstrate that hiSCAPs are multipotent and telomere length maintenance affects the differentiation capacity of stem cells. In summary, these results suggested that hiSCAPs with, however not limited to, the phenotypic characteristics of their parental cells may be promising seed cells for studies on cellular mechanisms and regenerative processes.

Cells are key factors in tissue engineering and bone growth strategy take advantage from the use of MSCs. PDLSCs combined with different biomaterials can promote the bone regeneration process to treat bone loss and ossification defects caused by aging or accidental or surgical trauma (Diomede et al., 2016). Gingiva-derived MSCs are also reported to be candidates for tissue engineering and cell-based therapy (Seo et al., 2004). SCAPs represent an early stem/progenitor cell type with good proliferative capacity and potential for osteogenic differentiation; therefore, they are preferable choices for bone tissue regeneration. We demonstrated the excellent osteogenic activity of established hiSCAPs, indicating that hiSCAPs may also play a big role in the bone regeneration field. Growth factors are also essential for the differentiation of cells into different lineages. BMP9 has been certified as one of the least characterized BMPs; however, it is the most potent osteoinductive factor among the BMP families (Cheng et al., 2003). Reportedly, BMP9 can effectively induce the osteogenic, adipogenic, and chondrogenic differentiation of MSCs (Cheng et al., 2003; Kang et al., 2009). In this study, our results confirmed that hSCAPs and hiSCAPs infected with AdBMP9 revealed higher ALP activity and more calcified deposits. ALP activity, known as an indicator of calcification, was also considered an index indicating a state of cells that could continue to grow and divide indefinitely (Inada et al., 2017). We also demonstrated that BMP9 effectively promoted osteogenic differentiation and subsequent mineralization of hSCAPs and hiSCAPs *in vivo* by ectopic bone formation assay. Furthermore, a study confirmed that telomerase can promote osteogenic differentiation of human BMSCs by up-regulating the expression of RNUX2, SP7, and OCN (Gronthos et al., 2003). In this study, *in vivo* and *in vitro* experiments verified that the osteogenic differentiation potential of telomerase-expressing cells was improved. These results support the application of hiSCAPs in the field of bone tissue engineering. We further explored the potential value of hiSCAPs as seed cells for studying the molecular mechanisms of osteogenic differentiation. BMP9 induces osteogenic differentiation by binding to two receptors. Among them, BMPRII has been confirmed as a functional type II TGF-β receptor for the osteogenic differentiation of MSCs induced by BMP9 (Wu et al., 2010). On the one hand BMPRII possesses a similar domain organization with other type II TGF-b receptors, namely, ActRII and ActRIIB, but on the other, it has a long C-terminal tail region which may contribute to the interaction with some proteins and regulation of the BMP pathway (Hassel et al., 2004). Moreover, compared with seven functional type I receptors, only ALK1 and ALK2 mutants could inhibit the osteogenic differentiation of MSCs *in vitro* and ectopic bone formation *in vivo*, which indicated the essential and specific role of ALK1 in BMP9-induced osteoinductive signaling (Luo et al., 2010; Song et al., 2013). In this study, we also demonstrated that BMP9 increased the expression of its osteogenic receptor BMPRII and ALK1 to induce the osteogenic differentiation of hSCAPs and hiSCAPs.

In general, theseresults support the potential use of hiSCAPs in tissue engineering. Many adult stem cells currently exist for regeneration and repair studies, such as bone marrow-derived MSCs, skin-derived iPSCs, and blood-derived HSCs; however, dental-derived stem cells are favored owing to their unique advantages. SCAPs are easily accessible, possess the potential for multiple differentiation, and display no tumorigenicity. More importantly, hiSCAPs maintain multipotential, can proliferate infinitely, and do not exhibit genomic instability. These results prove that hiSCAPs have potential in research and as therapeutics. Furthermore, *in vivo* experiments confirmed the safety of these cells in an animal model and opened possibilities for therapeutic applications in humans.

## Conclusion

In this study, we successfully immortalized hSCAPs with lentiviruses overexpressing hTERT and demonstrated that hiSCAPs possess the ability of self-renewal, unlimited proliferation without tumorigenicity, and multipotent differentiation, which supports the use of hiSCAPs for studies on cellular mechanisms and regenerative processes, and for applications in bone tissue engineering. In addition, our results provided evidence that BMP9 may be an efficacious bio-factor that enhances the osteogenic capacity in tissue engineering.

## Data Availability

The original contributions presented in the study are included in the article/supplementary material, further inquiries can be directed to the corresponding author.
